# Vitamin C improves the therapeutic potential of human amniotic epithelial cells in premature ovarian insufficiency disease

**DOI:** 10.1186/s13287-020-01666-y

**Published:** 2020-04-22

**Authors:** Shunyu Hou, Chenyue Ding, Han Shen, Chunfeng Qian, Qinyan Zou, Jiafeng Lu, Boxian Huang, Jichun Tan, Hong Li

**Affiliations:** 1 Department of Obstetrics and Gynecology, Affiliated Suzhou Hospital of Nanjing Medical University, Suzhou Municipal Hospital, Suzhou, 215002 China; 2 Center of Reproduction and Genetics, Affiliated Suzhou Hospital of Nanjing Medical University, Suzhou Municipal Hospital, Suzhou, 215002 China; 3 State Key Laboratory of Reproductive Medicine, Nanjing Medical University, Nanjing, 210029 China; 4 Reproductive Medical Center of Gynecology and Obstetrics Department, Shengjing Hospital of China Medical University, Shenyang, 110000 China; 5 Key Laboratory of Reproductive Dysfunction Diseases and Fertility Remodeling of Liaoning Province, Shenyang, 110000 China

**Keywords:** Human amniotic epithelial cell, Vitamin C, Premature ovarian insufficiency, Growth factors

## Abstract

**Background:**

Human amniotic epithelial cell (hAEC) transplantation holds great promise in treating premature ovarian insufficiency (POI). However, some deficient biological characteristics of hAECs restrict their application.

**Methods:**

Vitamin C (VC) was added to the culture media of hAECs for 2 weeks. Then, the proliferative ability, migration ability, pluripotency, and self-renewal of VC-treated hAECs (VC-hAECs) were determined. Next, hAECs and VC-hAECs were transplanted into the ovaries of cyclophosphamide (CTX)-induced POI model mice. The ovarian function of POI mice was evaluated after transplantation by counting follicle numbers and measuring the blood levels of AMH, E2, and FSH. The rescue effects of VC-hAECs and hAECs were unveiled by coculturing with CTX-damaged human ovarian granulosa cells (hGCs) and analyzing relative marker expression. Additionally, ovarian marker expression and transplant survival were detected in POI mice after transplantation to verify the beneficial effect of VC-hAECs. The cytokine profiles of VC-hAECs and hAECs were revealed by performing a cytokine array and an ELISA to show their paracrine function.

**Results:**

Our results indicated that VC promoted the proliferation, migration, pluripotency, and self-renewal of hAECs in vitro. The most effective concentration of VC was 50 μg/ml. After transplantation into the POI mouse model, VC-hAECs reversed ovarian function more powerfully than hAECs. Human granulosa cell marker expression in CTX-damaged hGCs was increased after coculture with VC-hAECs compared with hAECs. In the ovaries of the POI mice, ovarian marker expression was greater after VC-hAEC transplantation than after hAEC transplantation. VC-hAECs showed higher transplant survival than hAECs. Furthermore, VC-hAECs secreted more growth factors than hAECs.

**Conclusion:**

Treatment with VC promoted the proliferation, migration, self-renewal, and paracrine functions of hAECs. Additionally, VC elevated the therapeutic potential of hAECs in treating POI.

## Introduction

Premature ovarian insufficiency (POI), which is also referred to as premature ovarian failure (POF), is a reversible syndrome affecting the female population under the age of 40 [1]. Characterized by ovarian function cessation, POI has become one of the leading causes of infertility in China. Accessible treatments for POI, such as hormone replacement therapy and ovulation induction, are not satisfying. Therefore, recent studies have been focused on the search for alternative treatments, such as stem cell therapy.

Ovarian regeneration after stem cell therapy results from complex and unclear factors. Researchers have demonstrated that paracrine factors, including VEGF, HGF, IGF-1, and FGF2, mediate the repair of damaged ovaries [2, 3]. Others have suggested that stem cell-derived exosomes and exosomal microRNAs (miR-144-5p) inhibit ovarian cell apoptosis [4–6]. Recently, several studies noted that material transfer between host and donor cells accounts for the rescue effect of stem cell therapy [7, 8]. Additionally, we cannot rule out the possibility that transplanted stem cells may integrate into host ovaries, differentiate into ovarian cells, and replace the impaired cells of the recipients.

To date, various kinds of stem cells have been investigated to treat POI, including adipose-derived mesenchymal stem cells (hADSCs) [6], fetal liver mesenchymal stem cells [9], bone marrow mesenchymal stem cells (hBMSCs) 2 [10], human umbilical cord-derived mesenchymal stem cells (hUCMSCs) [11, 12], human amniotic epithelial cells (hAECs) [13, 14], human amniotic fluid stem cells [15], and human amniotic mesenchymal stem cells (hAMSCs) [16, 17]. Previous studies have indicated that human amnion-derived stem cells are easier, less invasive, and more cost-effective than to obtain hADSCs and hBMSCs because amniotic tissues are discarded after delivery [16, 18]. Moreover, hAECs exhibit low immunogenicity and no tumorigenicity, which make them an ideal candidate for regenerative medicine [13, 19]. However, the insufficient propagation ability and deficient paracrine function of hAECs has restricted their application in reversing ovarian dysfunction [16, 18].

Recently, to improve the efficacy of cell-based therapy, several solutions have been proposed, such as genetic engineering [20], scaffolds [21], and delivery system optimization [22]. The safety of genetically engineered cells is concerning due to transcriptome alterations and increased tumorigenicity. Scaffolds will support transplanted cells, but their degradation and immunogenicity do not put forth a solution. Additionally, the advancement for delivery system optimization is limited.

With small molecule compounds, cell characterizations can be regulated and modified in a nonimmunogenic, temporal, and standardized way. Vitamin C (VC) is known as a natural antioxidant. Studies have shown that VC can accelerate proliferation, promote self-renewal, and induce a pluripotent state in a variety of stem cells [23–25]. Supplementation with VC increased histone demethylase JARID1A and pluripotency markers (NANOG, SOX2, C-MYC, and KLF4) expression [24, 26–28]. VC has also been reported to improve proliferation, multidifferentiation potential, and extracellular matrix secretion of BMSCs [29–31]. However, the biological characterization alteration of hAECs after VC treatment has never been investigated.

For these purposes, in the present study, we modified the biological characterizations and elevated the therapeutic potential of hAECs with the small molecule, VC.

## Materials and methods

### Isolation and culture of human amniotic epithelial cells (hAECs)

The preparation and culture of hAECs as well as donor information were described previously [16]. In brief, human placental tissues from healthy women who were negative for HIV-I, hepatitis B, and C were collected after informed consent was signed. The chorion was washed with PBS and then separated to obtain the amniotic membrane. Then, membrane segments were dissolved in 0.25% trypsin/EDTA (Thermo Fisher Scientific, USA) for 45 min at 37 °C. Then, cells were seeded onto culture plates supplemented with 10% FBS-containing DMEM (Thermo Fisher Scientific, USA) and incubated at 37 °C in 5% CO_2_. Cells were passaged or analyzed after reaching confluence.

### Fluorescence-activated cell sorting (FACS)

Approximately 2 × 10^5^ hAECs, VC-hAECs, hGCs, or ovarian cells were isolated and washed with DPBS. Then, these 2 × 10^5^ cells were each added to a tube to undergo the following experiments. PE-conjugated or FITC-conjugated antibodies were incubated with the cells for 30 min in a dark room at room temperature (Antibody information, see Supplementary Table 1). Isotype controls were used to as negative controls. Before being tested by a fluorescence-activated cell sorter (Beckman, S. Kraemer Boulevard Brea, CA, USA), cells were washed and resuspended in 100 μl of PBS. Fixation and permeation buffers (BD, USA) were used before antibody incubation when intracellular proteins were detected. At least three assays were performed for each experiment.

### Wound healing assay

A culture insert (Ibidi, Germany) was employed to assay the cell migration ability of hAECs and VC-hAECs. Approximately 3 × 10^5^ hAECs were seeded onto the culture insert and cultured overnight. Then, a standardized wound of 500 μm was made using a sterile tweezer. Afterwards, PBS, 25 μg/ml VC, 50 μg/ml VC, or 100 μg/ml VC was added to the culture insert. Images were captured and analyzed at 0 h, 24 h, and 48 h after wounding. The percent wound recovery was analyzed using Wimscratch Quantitative Wound Healing Image Analysis (Wimasis Gmbh, Germany).

### Western blot (WB) assay

To detect the protein-level marker expression in hAECs and hGCs, cells were dissolved and dissociated in lysis buffer (Beyotime Biotechnology, China). After extracting the protein from the cells, 20 μg of protein was loaded onto 10% gels and fractioned via 10% or 20% SDS-PAGE. Then, primary antibodies were used to bind the protein after the separated proteins were electroblotted onto PVDF. The expression of the proteins was tested by incubating with secondary antibodies and detected with enhanced chemiluminescence. By analyzing the gray value of the band of interest with ImageJ software (National Institutes of Health, USA), the expression levels of the proteins were calculated. Each experiment was repeated at least three times. The information of the first antibody is listed in Supplementary Table 2.

### Premature ovarian insufficiency (POI) mouse model establishment

Female C57B6L/J mice aged 8 weeks were purchased from the Institute of Animal Research at Nanjing Medical University. The Ethics Committee of Nanjing Medical University (approval number: 20170480) approved this study. Mice were equally and randomly sorted into 4 groups: a normal group with no treatment (NG, *n* = 10), a POI group treated with CTX followed by the transplantation of PBS (POI, *n* = 10), an hAECs group treated with CTX following transplantation of hAECs (hAECs, *n* = 10), and a VC-hAECs group treated with CTX following transplantation of the VC-treated hAECs. CTX was administered by intraperitoneal injection at a concentration of 120 mg/kg.

### Cell transplantation

Probe DiO (Invitrogen, USA, Cat: D275) was employed to trace VC-hAECs and hAECs after transplantation. After labeling with DiO according to the manufacturer’s instructions, VC-hAECs and hAECs were dissolved in and washed with PBS before being resuspended in PBS at a volume of 10 μl. Two weeks after CTX injection, VC-hAECs and hAECs were transplanted into mouse ovaries through the caudal vein with microinjection needles by laparotomy. Mice in the POI group were injected with 10 μl of PBS as a control.

### Immunohistochemical staining

At 3, 7, and 14 days after cell transplantation, mice were sacrificed, and ovarian tissues were taken. Ovarian tissues were cut at a thickness of 5 μm and then fixed in 4% (w/v) paraformaldehyde (Sigma, USA). After being permeated with 0.1% Triton-X-100 for 5 min at 4 °C, ovarian sections were blocked with 4% BSA (Sigma, USA) for 1 h at room temperature. After three washes, ovarian sections were incubated with primary antibodies overnight at 4 °C. The primary antibodies against human-MVH (Abcam, USA, catalog number: ab13840) were used to label the ovarian cells. Next, mouse sections were washed with PBS three times after rewarming. A PE-conjugated secondary antibody was used to stain ovarian tissues by incubation in a dark room for 30 min at room temperature. Finally, ovarian sections were mounted using Hoechst-Fluoromount-G (Southern Biotech, USA) and viewed under a Nikon Eclipse E800 microscope.

### Alkaline phosphatase (AP) staining

The expression of AP at the surface of hAECs and VC-hAECs was measured with an AP staining kit (System Biosciences, Canada) following the manufacturer’s instructions. In brief, cells were washed with PBS before being fixed for 5 min at room temperature. Then, the fixing solution was removed, and the AP substrate solution was added to the culture plate. After incubation for 20 min at room temperature, staining solution was given to stop the reaction. Finally, the AP^+^ cells (blue stained) were observed with a light microscope.

### Hematoxylin and eosin (HE) staining and ovarian follicle count

At 8 weeks posttransplantation, the mice were euthanized. Then, the ovaries on both sides of each mouse were removed and fixed in 10% paraformaldehyde for 2 h at room temperature. Then, the ovaries were embedded in paraffin and sectioned at a thickness of 5 μm. Five sections were collected from each ovary. The ovarian structure and follicle phenotype are shown by staining with HE. Primordial follicles, primary follicles, secondary follicles, antral follicles, and total follicles were classified and calculated. To avoid recounting, each oocyte was counted once. This experiment was repeated at least three times.

### Cytokine array

Paracrine profiles of hAECs and VC-hAECs were measured by protein antibody array (RayBiotech, USA). Culture media from hAECs and VC-hAECs was collected and centrifuged at 13,000 rpm for 20 min at 2–8 °C before incubation with antibodies. Images were captured with an Axon GenePix laser scanner. Then, fluorescence intensity data were analyzed using RayBio Analysis Tool software.

### Enzyme-linked immunosorbent assay (ELISA)

To detect hormone levels in mice, the protein levels of serum E2, FSH, and AMH in mice were measured. At 0, 1, 2, 3, 4, and 5 weeks posttransplantation, blood samples (0.5 ml) were collected. Then, the blood samples were centrifuged at 4000 r/min for 10 min and the blood cells were discarded. When detecting the paracrine activity of selected cytokines in hAECs and VC-hAECs, culture media was collected and centrifuged at 4000 r/min for 10 min. The supernatant was isolated and detected by ELISA kits (Mybiosource, USA) following the manufacturer’s instructions. In brief, 50 μl of serum or culture medium was added to one test well, followed by incubation and washing. After adding Stop Solution, samples were immediately measured by a spectrophotometer (Varian Company, Australia). Each sample was detected at least three times.

### Statistical analysis

All experiments in this study were repeated at least 3 times. The values are shown as the mean ± SD. Before Scheffe’s *t* test (SPSS 17.0 software) was conducted, one-way ANOVA was performed. Probability values < 5% were considered significant.

## Results

### VC promoted the proliferation of hAECs

**Fig. 1:**
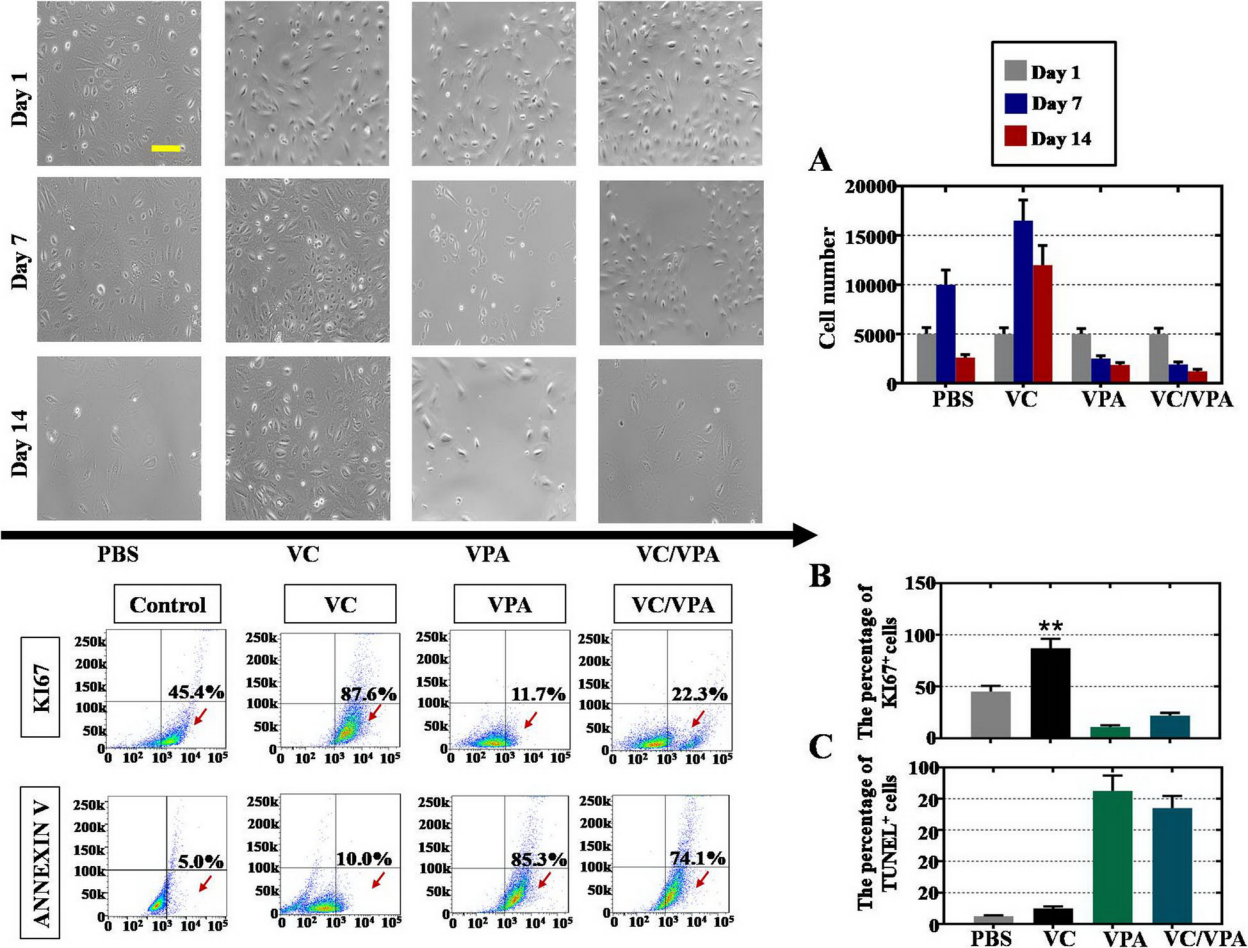
VC promoted proliferation and repressed apoptosis in hAECs. **a** Light images showing the morphologies and cell densities of hAECs at 1, 7, and 14 days after treatment with VC and/or VPA. The cell numbers are calculated and presented on the right. Scale bar = 20 μm. **b**, **c** FACS scatter plots showing KI67^+^ and TUNEL^+^ hAECs at 7 days after treatment with VC and/or VPA. The percent KI67^+^ and TUNEL^+^ cells are summarized on the right. hAECs were given PBS as a control. One-way ANOVA was performed followed by Scheffe’s *t* test, **p < 0.01, versus PBS group

First, the roles of VC and VPA (Valproic acid) in regulating the propagation and apoptosis of hAECs were preliminarily elucidated. PBS, 50 μg/ml VC, and/or 50 μg/ml VPA were added to the culture media of hAECs at passage 5 every 24 h. The light images and cell counting results in Fig. 1a show that the cell density of VC-treated hAECs was dramatically higher than that of PBS-treated hAECs 7 and 14 days after treatment. However, the cell densities of hAECs treated with only VPA and hAECs treated with both VC and VPA were lower than those of hAECs treated with PBS at 7 days. The FACS outcome 7 days after treatment verified these observations, and the percent KI67^+^ hAECs was significantly increased in VC-treated group compared with the PBS-treated group (Fig. 1b). The percent ANNEXIN V^+^ hAECs significantly and dramatically increased after treatment with only VPA and treatment with both VC and VPA compared with hAECs treated with only PBS (Fig. 1c). In summary, our results indicated that VC promoted hAEC proliferation and VPA induced hAEC apoptosis.

### VC facilitated cell migration and extended the life span of hAECs

**Fig. 2:**
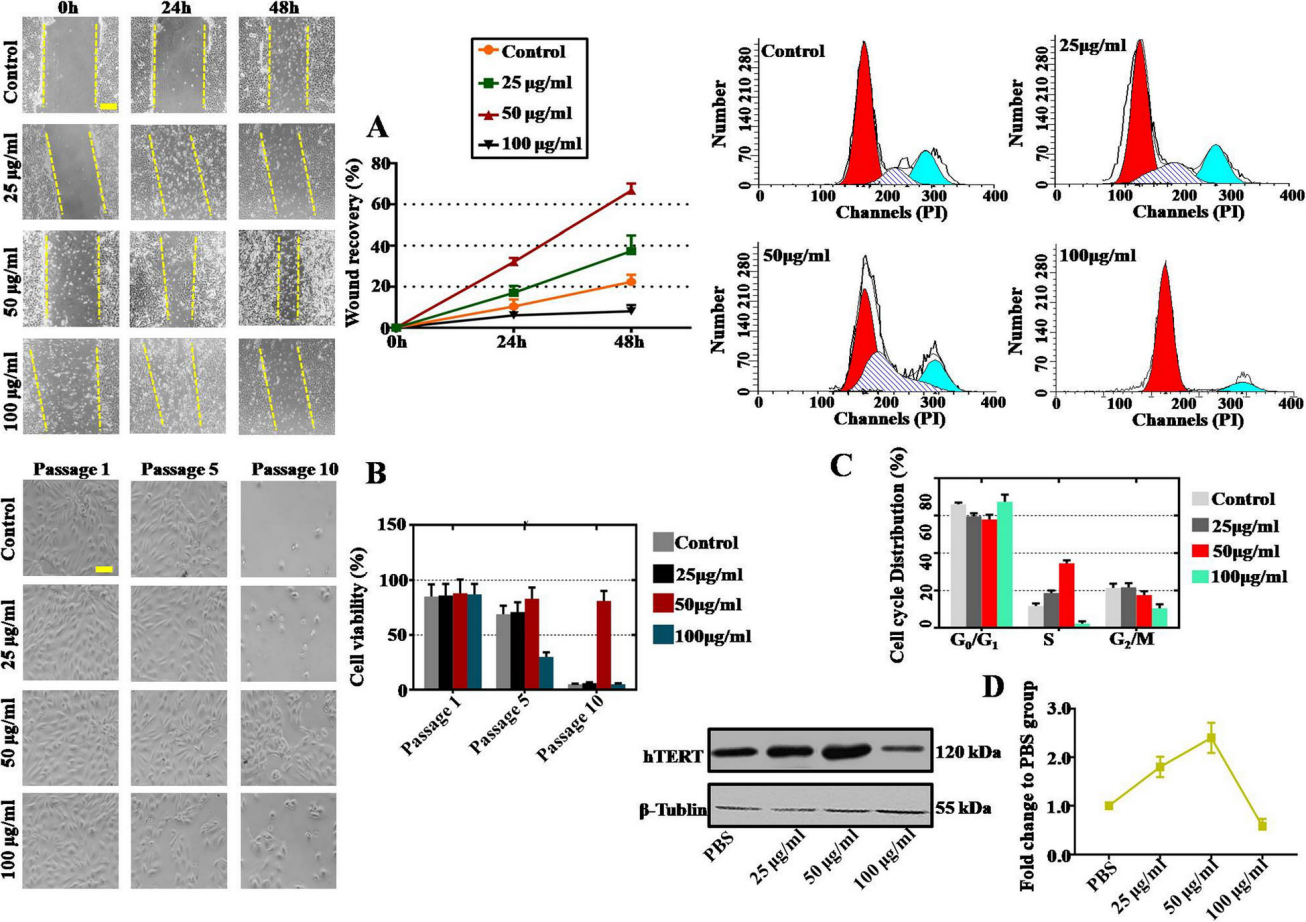
VC promoted the migration ability and extended the life span of hAECs. **a** Wound healing assay results showing the migratory ability of hAECs after treatment with 25, 50, and 100 μg/ml VC. The yellow dotted lines depict the boundaries of the wound. **b** hAEC viabilities measured at passage 1 (P1), P5, and P10 after treatment with 25, 50, and 100 μg/ml VC from P1. Cell viability assay results at different passages are summarized on the right. Cell viability was determined by trypan blue. Scale bar = 20 μm. **c** Cell cycle analysis of hAECs at passage 5 showing G1 phase, S phase, and G2 phase fractions after treatment with 25, 50, and 100 μg/ml VC. The percent hAECs in the G_0_/G_1_, S, and G_2_/M phases are summarized below. **d** WB results indicating the protein levels of hTERT in hAECs at passage 5 after treatment with 25, 50, and 100 μg/ml VC. PBS was added to the culture media of the hAECs as a control

Then, the optimal effective concentration of VC was evaluated by adding PBS, 25 μg/ml VC, 50 μg/ml VC, or 100 μg/ml VC to the culture media of hAECs. Wound healing assay results illustrated that cell migration was dramatically elevated in 50 μg/ml VC-treated hAECs at 24 h and 48 h posttreatment (Fig. 2a). Moreover, trypan blue staining results showed that the percent viable hAECs at P10 was significantly and dramatically increased after administration of 50 μg/ml VC (Fig. 2b). By analyzing the cell cycle of hAECs at P10, we found that the percentage of cells in the S phase increased the most in the 50 μg/ml VC-treated hAECs (Fig. 2c). The WB results also showed the highest expression level of telomerase marker (hTERT) in 50 μg/ml VC-treated hAECs (Fig. 2d). Taken together, the proliferation, migration, and self-renewal of hAECs was greatly facilitated after VC treatment. Among the three doses of VC, 50 μg/ml showed the most effective outcomes.

### VC promoted pluripotency marker expression levels in hAECs

**Fig. 3:**
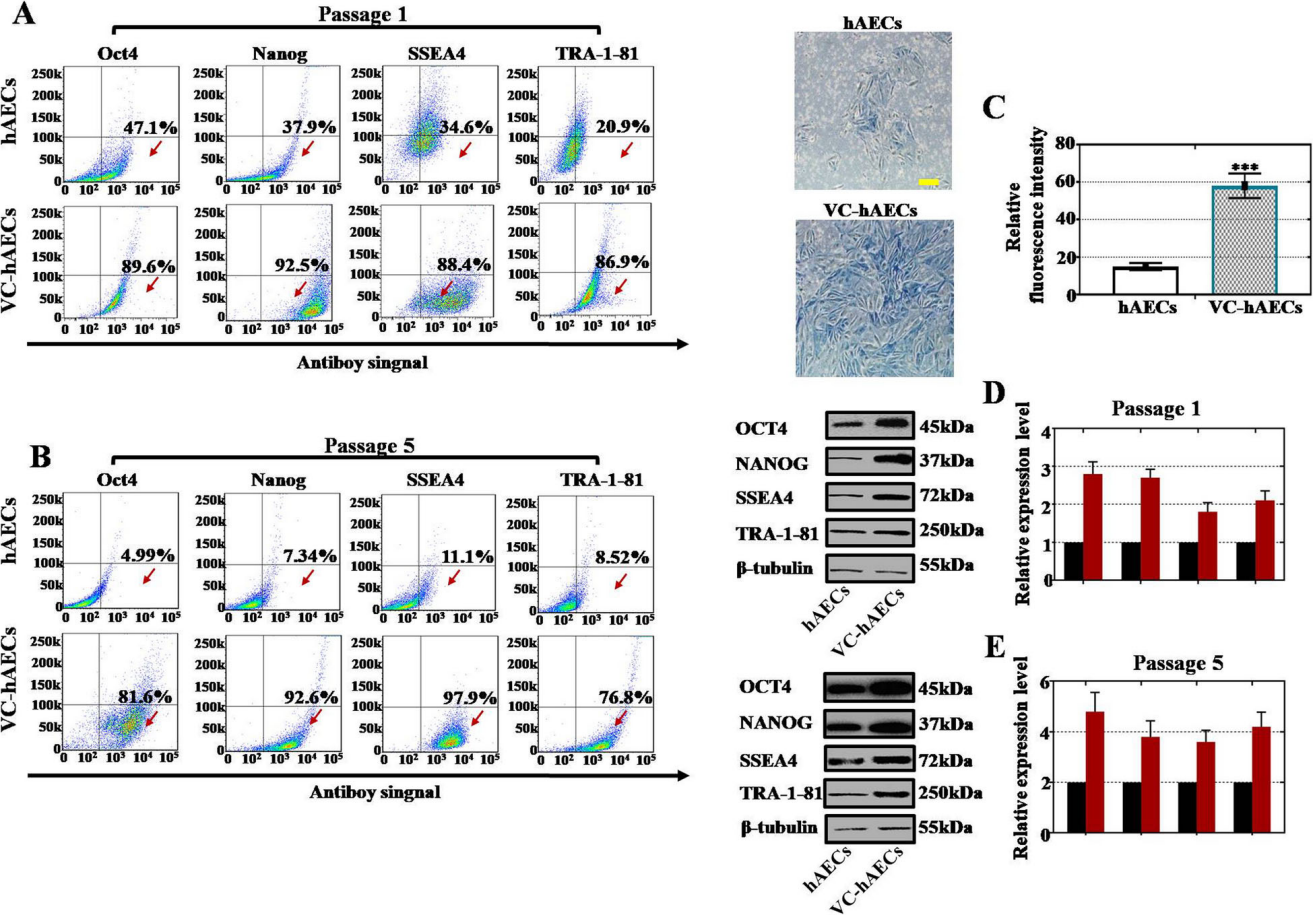
VC treatment promoted pluripotency marker expression of hAECs. **a**, **b** FACS results illustrating the percent OCT4^+^, NANOG^+^, SSEA4^+^, and TRA-1-81^+^ hAECs and VC-hAECs at passage 1 and passage 5. **c** Alkaline phosphatase (AP) staining images of hAECs and VC-hAECs at passage 5. The relative fluorescence intensity was measured and is presented on the right. Paired *t* test, ***p < 0.001, versus hAEC group. **d**, **e** Protein-level assay results showing pluripotency marker (OCT4, NANOG, SSEA4, and TRA-1-81) expression levels in hAECs and VC-hAECs at passage 1 and passage 5. VC-hAECs = hAECs treated with VC

To unveil the role of VC in regulating the pluripotency of hAECs, the expression of pluripotency markers in hAECs and hAECs treated with 50 μg/ml VC (VC-hAECs) was detected. According to our FACS results, 50 μg/ml VC treatment greatly increased the percentages of OCT4-, NANOG-, SSEA4-, and TRA-1-81-positive cells in hAECs at P1 (increased from 4.99%, 7.34%, 11.1%, and 8.52%, respectively, to 81.6%, 92.6%, 97.9%, and 76.8%, respectively; Fig. 3a) and P5 (increased from 4.99%, 7.34%, 11.1%, and 8.52%, respectively, to 81.6%, 92.6%, 97.9%, and 76.8%, respectively; Fig. 3b). Using an enzymatic assay and a colorimetric method, our results showed that the expression of alkaline phosphatase (AP), a universal pluripotent marker for all types of pluripotent stem cells, was significantly elevated in VC-hAECs compared with hAECs (Fig. 3c). The WB results also demonstrated significantly upregulated expression of pluripotency markers (OCT4, NANOG, SSEA4, and TRA-1-81) in VC-hAECs compared with hAECs at P1 (Fig. 3d) and P5 (Fig. 3e). In addition, western blot assay was employed to test the protein level of ectoderm (Sox1, Nestin), mesoderm (T, CD31), and endoderm (Sox17, AFP). Our results indicated that vitamin C improved the protein level of ectoderm, mesoderm, and endoderm more powerfully than without vitamin C treatment (Supplementary Fig. 1). Altogether, our results demonstrated that VC promoted hAEC pluripotency.

### Ovarian function was more powerfully rescued in the VC-hAEC-transplanted POI mouse model than in the hAEC-transplanted POI mouse model

**Fig. 4:**
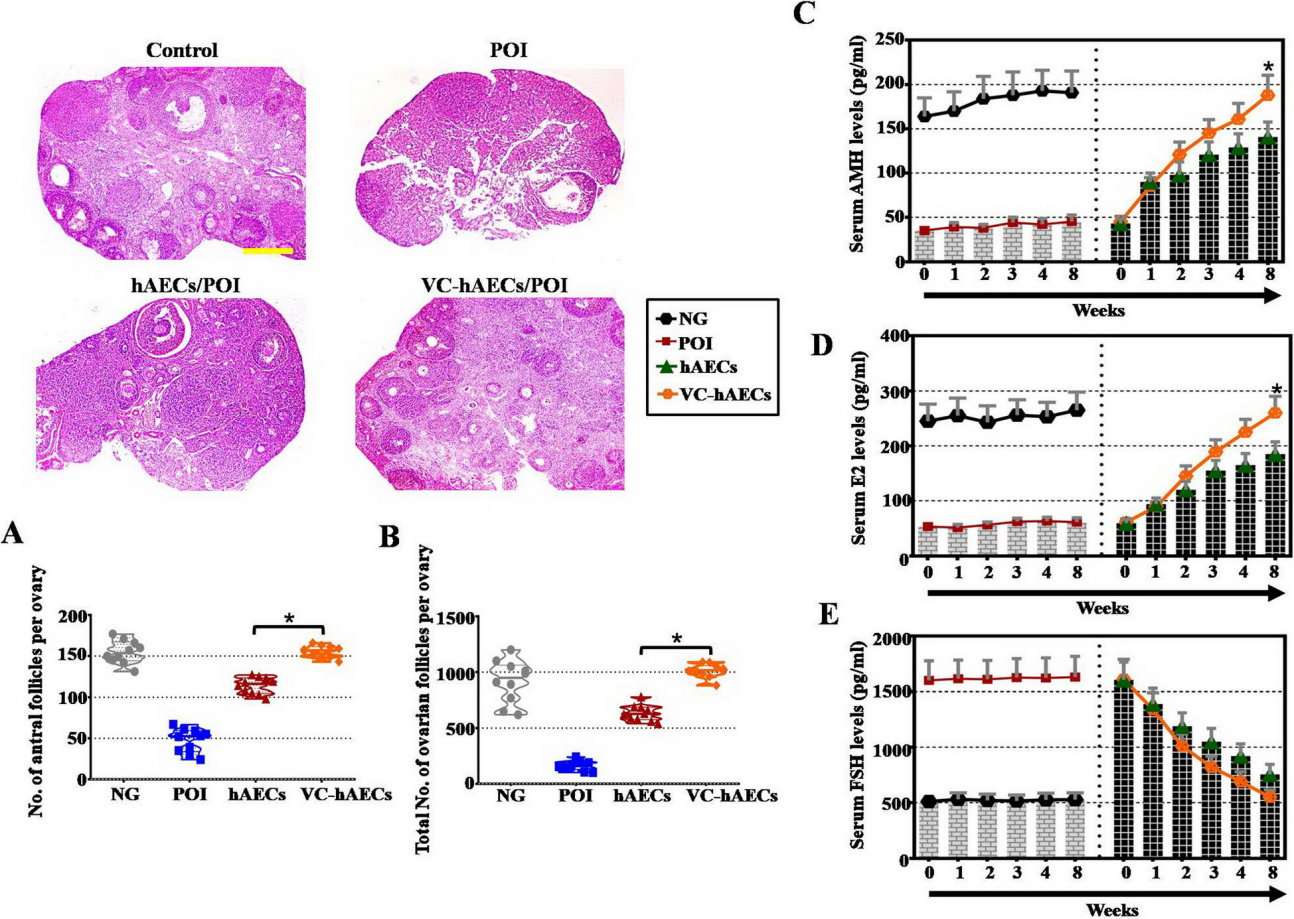
VC-hAEC transplantation showed a more beneficial rescue effect than hAECs on ovarian function in the POI mouse model. **a**, **b** HE-stained ovarian sections of a CTX-induced POI mouse model showing follicle phenotypes. The numbers of antral and overall follicles were calculated and are presented below. The corpus luteum (CF), primordial follicles (PF), primary follicles (1F), secondary follicles (2F), and antral follicle (AF) are indicated by red arrows. Scale bar = 100 μm. **c**–**e** Blood levels of AMH, E2, and FSH in a CTX-induced POI mouse model after transplantation of hAECs and VC-hAECs. One-way ANOVA was performed followed by Scheffe’s *t* test, *p < 0.05, versus hAECs group. NG = normal group with wild-type mice, POI = CTX-induced POI model mice, hAECs = CTX-induced POI model mice transplanted with hAECs, VC-hAECs = CTX-induced POI model mice transplanted with VC-treated hAECs

PBS, 2 × 10^5^ VC-hAECs, or hAECs were injected into the ovaries of cyclophosphamide (CTX)-induced POI mice to further elucidate the therapeutic potential of VC-hAECs to treat POI. The therapeutic effects of VC-hAECs and hAECs were assessed by counting follicles and measuring hormone levels. Primordial, primary, secondary, and antral follicles can be observed in the ovaries of VC-hAEC-transplanted POI mice (Fig. 4). Our data indicated that the numbers of antral and total follicles were significantly increased in the ovaries of VC-hAEC-transplanted POI mice compared with hAEC-transplanted POI mice and PBS-injected POI mice at 8 weeks posttransplantation (Fig. 4a, b). The serum hormone levels of E2 and AMH during the 8-week follow-up significantly and progressively increased in both hAEC- and VC-hAEC-transplanted POI mice compared with the PBS-injected POI mice. Furthermore, the serum levels of E2 and AMH at 8 weeks posttransplantation were significantly higher in VC-hAEC-transplanted POI mice than in hAEC-transplanted POI mice (Fig. 4c, d). At 8 weeks postoperation, the serum level of FSH was significantly reduced in the VC-hAEC-transplanted group compared with the hAEC-transplanted group (Fig. 4e). Collectively, our data indicated that VC enhanced the therapeutic potential of hAECs in a POI mouse model.

### hGC marker expression levels were elevated after coculture with VC-hAECs

**Fig. 5:**
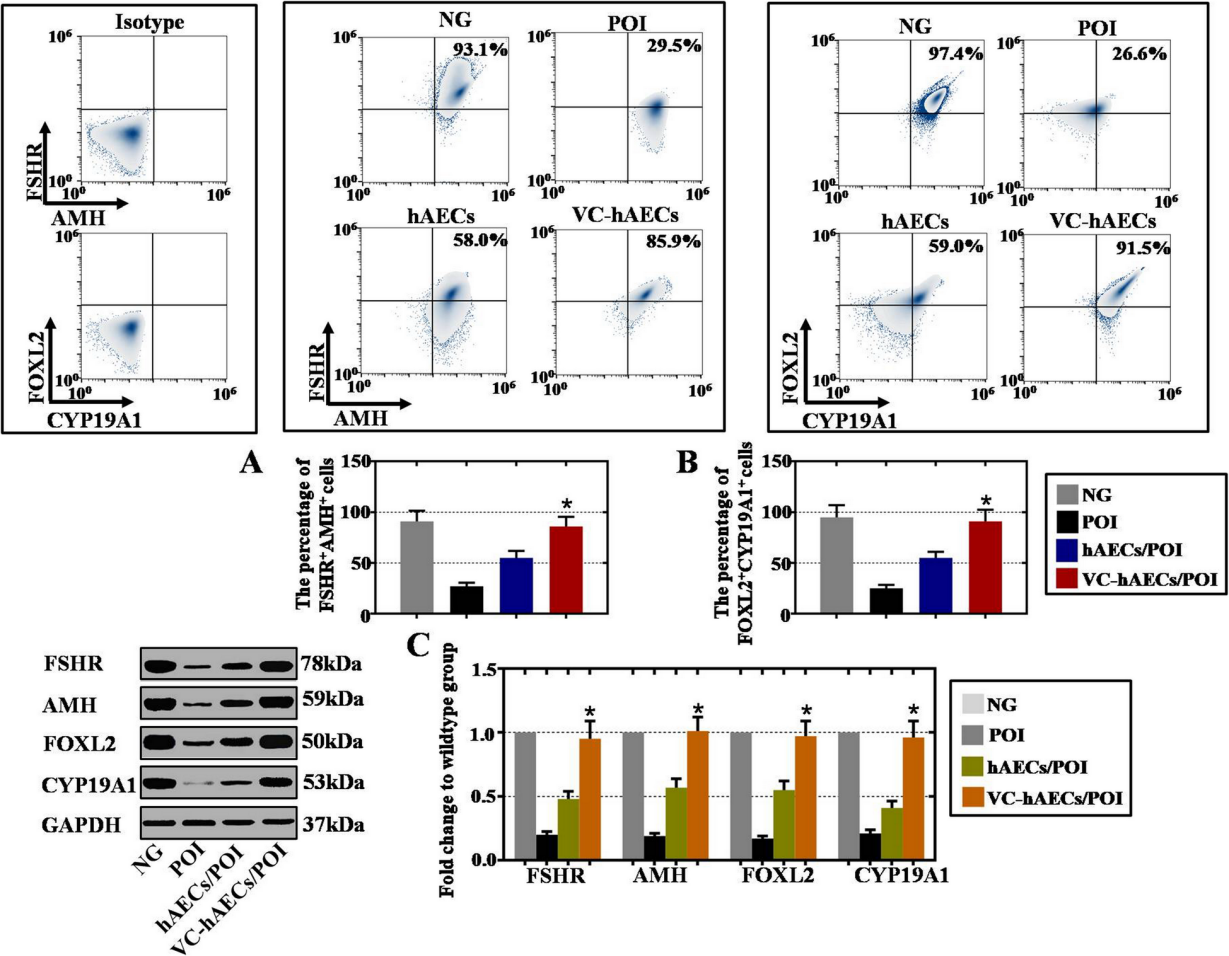
VC-hAECs elevated CTX-damaged hGC marker expression more powerfully than hAECs. **a**, **b** FACS results showing the percent of FSHR^+^AMH^+^ cells and FOXL2^+^CYP19A1^+^ cells in CTX-damaged hGCs after coculturing with hAECs and VC-hAECs. **c** Protein level analysis of hGC marker (FSHR, AMH, FOXL2, CYP19A1) expression in CTX-damaged hGCs after coculturing with hAECs and VC-hAECs. One-way ANOVA was performed followed by Scheffe’s *t* test, *p < 0.05, versus hAECs/POI group. NG = normal group with PBS-treated normal hGCs, POI = CTX-damaged hGCs, hAECs/POI = CTX-damaged hGCs cocultured with hAECs, VC-hAECs/POI = CTX-damaged hGCs cocultured with VC-treated hAECs

A human ovarian granulosa cell line (GC1a) was donated by Dr. Hitoshi Okamura at Kumamoto University and treated with CTX to establish a POI model in vitro (POI group). After coculture with hAECs (hAECs/POI group) and VC-hAECs (VC-hAECs/POI group), the expression of hGC markers (FSHR, AMH, FOXL2, CYP19A1) was examined. As shown in Fig. 5a, b, the FACS results showed that the percent FSHR^+^AMH^+^ and FOXL2^+^CYP19A1^+^ hGCs in the VC-hAECs/POI group (85.9% and 91.5%, respectively) were significantly higher than those of the hAECs/POI group (58.0% and 59.0%) and POI group (29.5% and 26.6%). This result was further validated by WB analysis as shown in Fig. 5c: the protein levels of FSHR, AMH, FOXL2, and CYP19A1 were significantly elevated in the VC-hAECs/POI group (4-fold, 5-fold, 4-fold, and 4-fold increase compared with the POI group, respectively) when compared with the hAECs/POI group (2-fold, 2-fold, 2-fold, and 2-fold increase compared with the POI group, respectively). Therefore, VC was demonstrated to elevate the restorative effect of hAECs in treating CTX-damaged hGCs.

### Ovarian marker expression levels were greater in the ovaries of the VC-hAEC-transplanted mice than in the hAEC-transplanted POI mice model

**Fig. 6:**
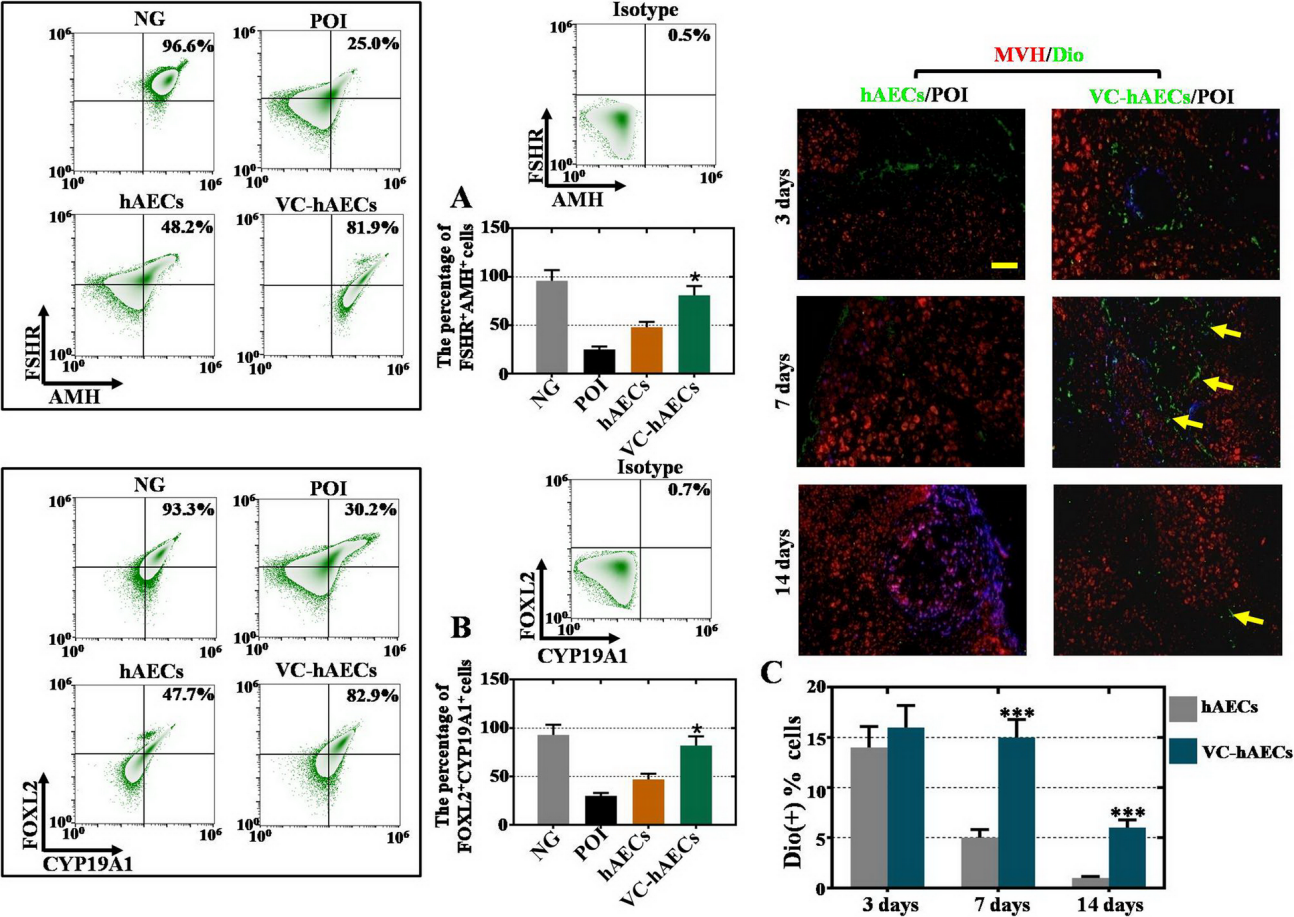
VC-hAEC transplantation showed a more powerful effect than hAECs on preserving ovarian marker expression in CTX-damaged ovaries. **a**, **b** FACS results showing the percent FSHR^+^AMH^+^ and FOXL2^+^CYP19A1^+^ ovarian cells in the CTX-induced POI mouse model. One-way ANOVA was performed followed by Scheffe’s *t* test, *p < 0.05, versus hAEC group. **c** Fluorescence images of mouse ovarian sections at 3, 7, and 14 days after transplantation of hAECs and VC-hAECs. CTX-damaged ovarian cells were stained with MVH (red). Yellow arrows indicate transplanted hAECs or VC-hAECs. Transplanted hAECs and VC-hAECs were labeled by DiO. Scale bar = 20 μm. NG = normal group with ovarian cells from wild-type mice, POI = ovarian cells from CTX-induced POI model mice, hAECs = ovarian cells from CTX-induced POI model mice transplanted with hAECs, VC-hAECs = ovarian cells from CTX-induced POI model mice transplanted with VC-treated hAECs

Then, ovarian transplantations of DiO-labeled VC-hAECs or hAECs were conducted in CTX-induced POI mice, followed by detection of the expression of ovarian markers (FSHR, AMH, FOXL2, CYP19A1) and analyzation of transplant survival. According to the FACS results, the percent FSHR^+^AMH^+^ and FOXL2^+^CYP19A1^+^ in ovarian cells in VC-hAEC-transplanted POI mice (81.9% and 92.9%, respectively) were significantly greater than hAEC-transplanted POI mice (48.2% and 47.7%) and POI mice (25.0% and 30.2%, Fig. 6a, b). At 3, 7, and 14 days posttransplantation, POI mouse ovaries were sectioned and stained for the ovarian marker MVH. Immunofluorescence images at 3 days showed similar DiO intensities (green) in ovarian tissues between the groups. At 7 days posttransplantation, more DiO-labeled VC-hAECs were observed than DiO-labeled hAECs in ovarian tissues. At 14 days posttransplantation, the DiO-labeled hAECs were almost all cleared, while DiO-labeled VC-hAECs could still be observed (Fig. 6c). In summary, VC was demonstrated to enhance the transplantation survival of hAECs and promote the rescue effect of hAECs in preserving CTX-damaged ovaries.

### VC elevated the paracrine activity of hAECs

**Fig. 7:**
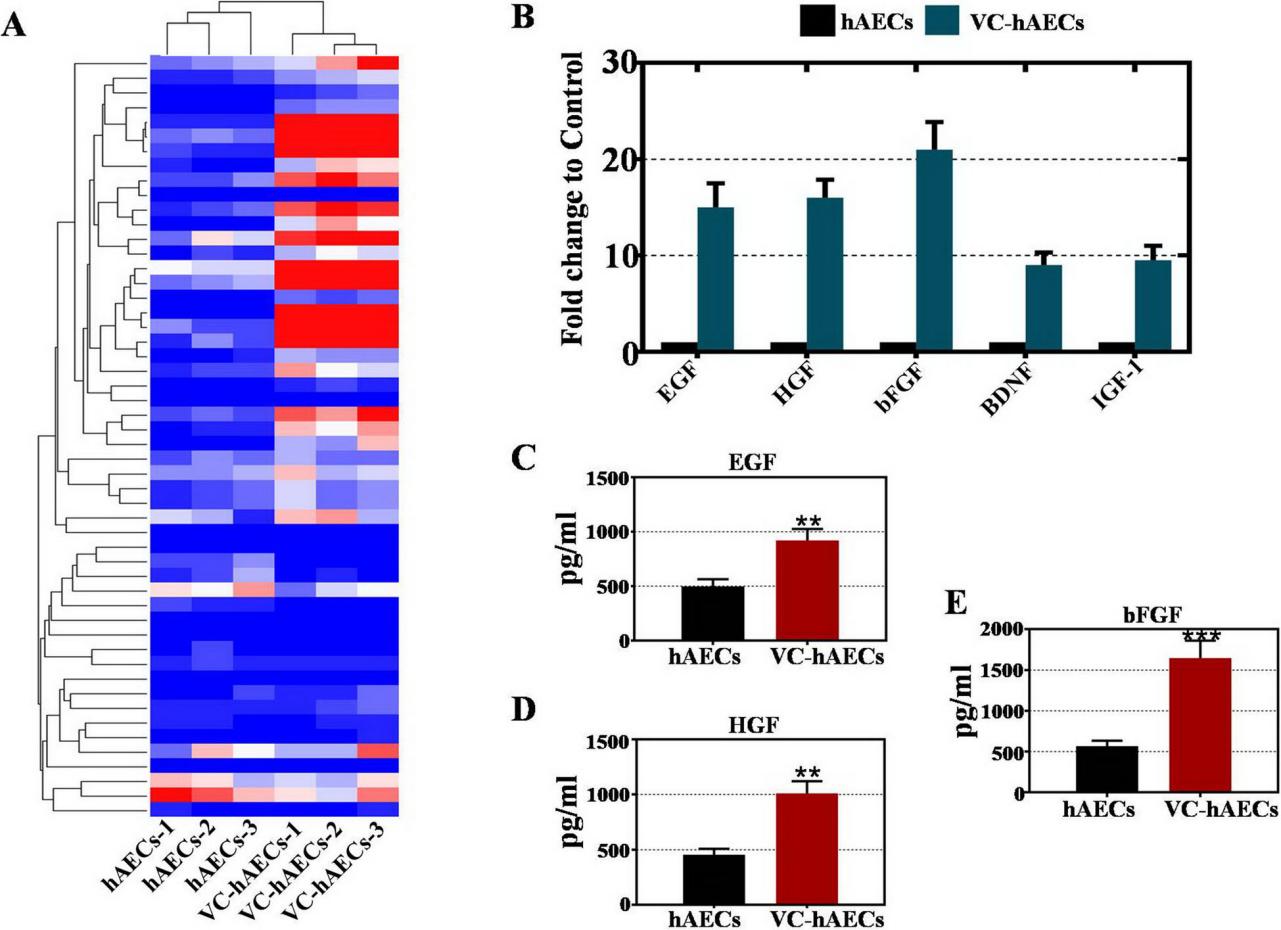
VC treatment elevated the paracrine function of hAECs. **a** Heatmap showing the cytokine profiles of hAECs and VC-hAECs. **b** Cytokine array results illustrating the paracrine activity of selective secreted cytokines (EGF, HGF, bFGF, BDNF, and IGF-1) in hAEC and VC-hAEC culture media. **c**–**e** ELISA results showing the secreted protein levels of EGF, HGF, and bFGF in hAECs and VC-hAECs culture media. VC-hAECs = hAECs treated with VC. **p < 0.01, ***p < 0.001, versus hAEC group

To unveil the paracrine function of cytokines in VC-hAECs, the culture media from VC-hAECs and hAECs were collected and underwent cytokine array analysis. The heatmap in Fig. 7a shows the cytokine profile of VC-hAECs and hAECs. The secretion of growth factors in hAECs was generally upregulated after VC treatment. The protein levels of secreted EGF, HGF, and bFGF from VC-hAECs were all over 10-fold higher than from the hAECs (Fig. 7b). ELISA results verified our previous findings and showed that the secreted protein levels of EGF, HGF, and bFGF from VC-hAECs were significantly increased compared with those from hAECs (Fig. 7c–e).

## Discussion

The present study is the first to report that supplementation with VC in vitro could promote proliferation and suppress apoptosis in hAECs. However, VPA supplementation did the opposite (Fig. 1). In accordance with previous studies, VC treatment was demonstrated to increase the proliferation rate in various kinds of mesenchymal stem cells [29, 32]. VPA was reported to accelerate the proliferation and differentiation of neural stem cells but inhibit the propagation of mesenchymal stem cells and epithelial cells [33–35].

Migration and homing ability is critical for hAEC-based therapy because these cells are expected to migrate to injury tissues [36]. Previous study demonstrated that the hAECs were found to migrate along nerve fibers in corpus callosum in the treatment of neurological diseases after transplantation [37]. Our results show that the migration ability of hAECs was highly facilitated by 50 μg/ml VC (Fig. 2a). Various factors influence the migration and homing efficiency in MSC, such as cell aging and culture conditions [36]. In our study, cell cycle progression was also promoted by 50 μg/ml VC, with an increased percentage in the S phase (Fig. 2c). Previous studies indicated that VC promoted the cell cycle by upregulating cyclin E1 and CDK2 and downregulating p53 and p21 [32]. The life span and telomere expression of hAECs was extended by 50 μg/ml VC (Fig. 2b, d). To sum up, the optimum working concentration of VC was found to be 50 μg/ml (Fig. 2). This may be attributed to the antioxidant role of VC [38]. Consistent with our findings, VC blocked the aberrant self-renewal of human hematopoietic stem cells [39]. In addition, we found that 100 μg/ml VC is toxic to hAECs. Others have also suggested that a high dose of VC could inhibit cell proliferation [40–42].

The expression of pluripotency markers in hAECs was greatly increased after VC treatment (Fig. 3). Previous studies have highlighted the upregulated SOX2 and OCT4 expression after adding VC [28]. Here, we showed that expression of NANOG, SSEA4 and TRA-1-81 was also activated by VC. Researchers suggested that VC promoted pluripotency in induced pluripotent stem cell (iPSC) reprogramming by inhibiting p53 and p21 expression [25] and restoring H3 lysine 4 trimethylation at Dlk1-Dio3 loci [43]. Others have indicated that VC induced a pluripotent state in embryonic stem cells by inducing microRNA-143 expression [23].

Finally, we observed a more powerful rescue effect of VC-treated hAECs (VC-hAECs) compared with hAECs in treating POI disease (Figs. 4, 5, and 6). First, ovarian function was improved in VC-hAEC-transplanted POI mice compared with the therapeutic effect of hAECs (Fig. 4). Second, after coculture with CTX-damaged hGCs, hGC marker expression was greater in the VC-hAEC group than in the hAEC group (Fig. 5). Third, in the ovaries of POI mice, ovarian markers were more highly expressed in the VC-hAEC transplanted group than in the hAEC transplanted group. Furthermore, transplanted VC-hAECs survived much longer than hAECs after ovary injection (Fig. 6). All these data elucidated the powerful role of VC in enhancing the therapeutic effects of hAECs. Whether VC-hAEC transplantation would achieve better outcomes than other graft resources (for example, hBMSCs, hADMSCs, or hUCMSCs) in cell-based therapy of POI needs further investigation.

By indirect coculture, VC-hAECs were found to more powerfully restore CTX-damaged hGCs compared with hAECs (Fig. 5). This result suggested that VC-hAECs showed therapeutic effects in a noncontact manner. In support of our findings, recent studies have suggested the paracrine mechanism of mesenchymal stem cell therapy [44]. Our previous study also demonstrated that hepatocyte growth factor (HGF) and epidermal growth factor (EGF) secreted from hAMSCs play a critical role in reversing ovarian aging [17]. In this study, VC-hAECs secreted more trophic factors than hAECs, including HGF and EGF (Fig. 7). Future studies will answer the question of whether the beneficial therapeutic effect of VC-hAECs is due to paracrine function or mediated by extracellular vesicles.

In conclusion, this study demonstrated that VC could increase the proliferation and migration ability and promote pluripotency marker expression and paracrine function of hAECs in vitro. Furthermore, the therapeutic potential of hAECs in preserving ovarian cells and rescuing ovarian function was greatly increased after VC treatment. Our research shed light on transplant modification by small molecules and suggested VC-treated hAECs as a beneficial graft resource in treating POI.

## Supplementary information

**Additional file 1: Figure S1.** VC treatment improved differentiation potential of hAECs into three germ cell lineage. After VC treatment, the protein level of ectoderm (Sox1, Nestin), mesoderm (T, CD31), endoderm (Sox17, AFP) were elevated.

**Additional file 2: Table S1.** Information regarding the flow cytometry antibodies.

**Additional file 3: Table S2.** Information regarding the western blot antibodies.

## Data Availability

All the data generated or analyzed during this study are included in this published article.

## Abbreviations

hAEC: Human amniotic epithelial cell
VC-hAEC: VC-treated human amniotic epithelial cell
hAMSC: Human amniotic mesenchymal stem cell
VC: Vitamin C
VPA: Valproic acid
hGC: Human ovarian granulosa cell
POF: Premature ovarian failure
hADSCs: Adipose-derived mesenchymal stem cells
hBMSCs: Human bone marrow mesenchymal stem cells
hUCMSCs: Human umbilical cord-derived mesenchymal stem cells
hTERT: Human *telomerase* reverse transcriptase
IVF: In vitro fertilization
CTX: Cyclophosphamide
POI: Premature ovarian insufficiency
iPSC: Induced pluripotent stem cells
HGF: Hepatocyte growth factor
EGF: Epidermal growth factor
AP: Alkaline phosphatase
