# Supplementary figures and images for: Vitamin C improves the therapeutic potential of human amniotic epithelial cells in premature ovarian insufficiency disease
Source: Stem Cell Res Ther. 2020 Apr 22;11:159. doi: 10.1186/s13287-020-01666-y (PMC7178972; doi:10.1186/s13287-020-01666-y)

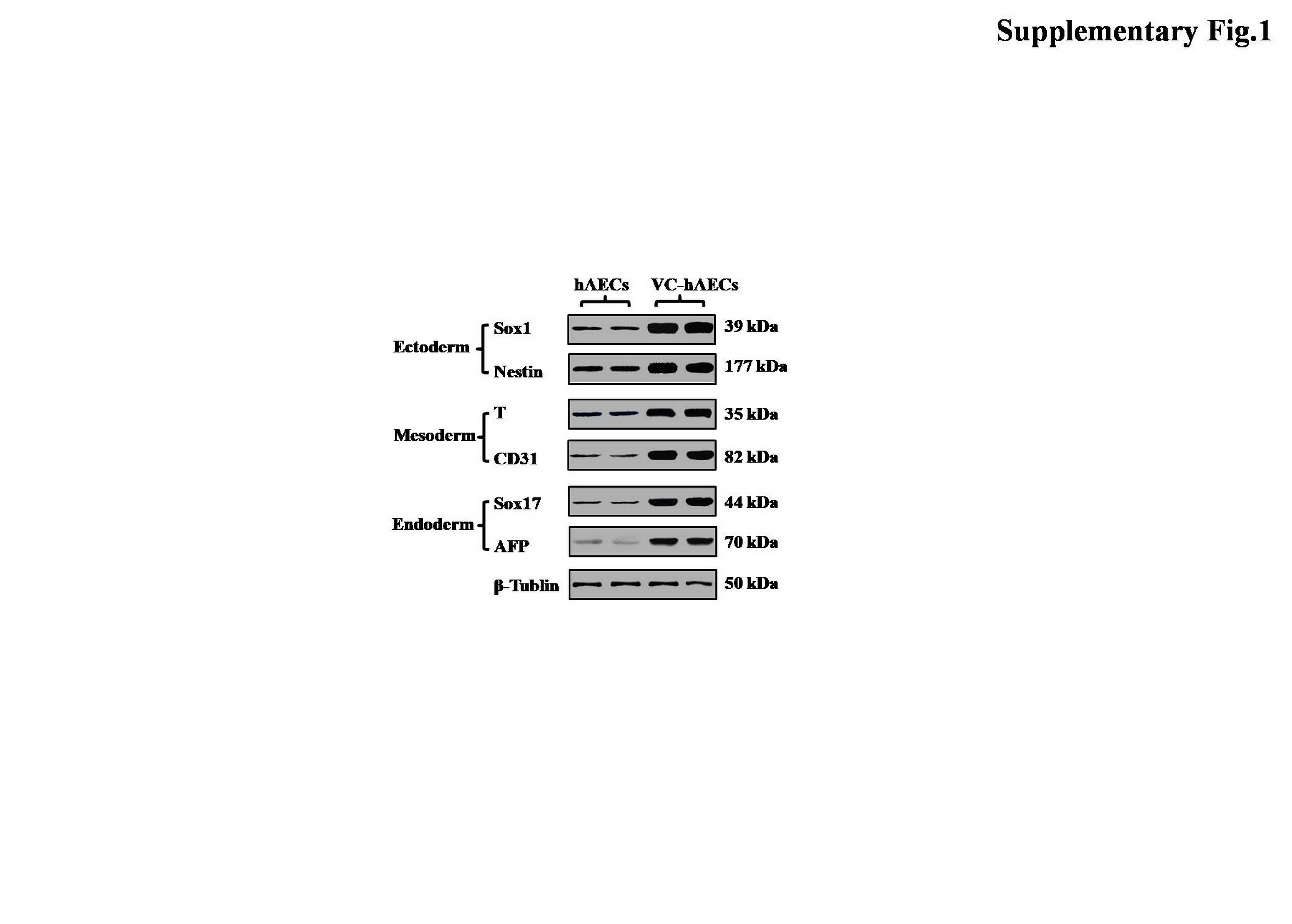

Supplement: Supplementary file 1 — Additional file 1: Figure S1. VC treatment improved differentiation potential of hAECs into three germ cell lineage. After VC treatment, the protein level of ectoderm (Sox1, Nestin), mesoderm (T, CD31), endoderm (Sox17, AFP) were elevated. [file 13287_2020_1666_MOESM1_ESM.tif]
