# Supplementary material for: Vitamin C improves the therapeutic potential of human amniotic epithelial cells in premature ovarian insufficiency disease
Source: Stem Cell Res Ther. 2020 Apr 22;11:159. doi: 10.1186/s13287-020-01666-y (PMC7178972; doi:10.1186/s13287-020-01666-y)
Supplement: Supplementary file 2 — Additional file 2: Table S1. Information regarding the flow cytometry antibodies. [file 13287_2020_1666_MOESM2_ESM.doc]

**Table S1.** Information regarding the flow cytometry antibodies.

| **Antibody** | **Vender, Catalog** | **Concentration** | **Country** |
| --- | --- | --- | --- |
| OCT4 | Abcam, ab181557 | 1:100 | USA |
| NANOG | Abcam, ab21624 | 1:60 | USA |
| SSEA4 | Abcam, ab16287 | 1.0 μl/Test | USA |
| TRA-1-81 | Thermo Fisher, 14-8883-82 | 0.5 μl/Test | USA |
| FSHR | Proteintech, 22665-1-AP | 0.2 µg/Test | USA |
| AMH | Invitrogen, PA5-26938 | 1:25 | USA |
| FOXL2 | NOVUS, NBP2-70013AF488 | 1:50 | USA |
| CYP19A1 | Abcam, ab215443 | 1:100 | USA |
| KI67 | BioLegend, 350504 | 2.5 μl/ Test | USA |
| ANNEXIN V | BD, 556420 | 0.5 μg/ml/Test | USA |
