# Supplementary material for: Vitamin C improves the therapeutic potential of human amniotic epithelial cells in premature ovarian insufficiency disease
Source: Stem Cell Res Ther. 2020 Apr 22;11:159. doi: 10.1186/s13287-020-01666-y (PMC7178972; doi:10.1186/s13287-020-01666-y)
Supplement: Supplementary file 3 — Additional file 3: Table S2. Information regarding the western blot antibodies. [file 13287_2020_1666_MOESM3_ESM.doc]

**Table S2.** Information regarding the western blot antibodies.

| **Antibody** | **Vender, Catalog** | **Concentration** | **Country** |
| --- | --- | --- | --- |
| hTERT | Abcam, ab32020 | 1/1000 | USA |
| OCT4 | Abcam, ab181557 | 1/1000 | USA |
| NANOG | Abcam, ab21624 | 1/200 | USA |
| SSEA4 | Abcam, ab16287 | 15 µg/ml | USA |
| TRA-1-81 | Abcam, ab16289 | 5 µg/ml | USA |
| FSHR | Proteintech, 22665-1-AP | 1/500 | USA |
| AMH | Invitrogen, PA5-26938 | 1:1000 | USA |
| FOXL2 | NOVUS, NBP2-70013AF488 | 1:500 | USA |
| CYP19A1 | Invitrogen, MA5-33132 | 1:1000 | USA |
| GAPDH | NOVUS, NB300-221 | 1/1000 | USA |
